# Transformation of an Olfactory Placode-Derived Cell into One with Stem Cell Characteristics by Disrupting Epigenetic Barriers

**DOI:** 10.1177/21524971251359000

**Published:** 2025-08-12

**Authors:** Ghazia Abbas, Rutesh Vyas, Joyce C. Noble, Brian Lin, Robert P. Lane

**Affiliations:** ^1^ Department of Molecular Biology and Biochemistry, Wesleyan University, Middletown, Connecticut, USA.; ^2^ Department of Developmental, Molecular, and Chemical Biology, Tufts University School of Medicine, Boston, Massachusetts, USA.

**Keywords:** epigenetics, differentiation, plasticity, olfactory, chromatin, reprogramming

## Abstract

The mammalian olfactory neuronal lineage is regenerative, and accordingly, maintains a population of pluripotent cells that replenish olfactory sensory neurons and other olfactory cell types during the life of the animal. Moreover, in response to acute injury, the early transit amplifying cells along the olfactory sensory neuronal lineage are able to de-differentiate to shift resources in support of tissue restoration. In order to further explore plasticity of various cellular stages along the olfactory sensory neuronal lineage, we challenged the epigenetic stability of olfactory placode-derived cell lines that model immature olfactory sensory neuronal stages. We found that perturbation of the *Ehmt2* chromatin modifier transformed the growth properties, morphology, and gene expression profiles toward states with several stem cell characteristics. This transformation was dependent on continued expression of the large T-antigen, and was enhanced by Sox2 over-expression. These findings may provide momentum for exploring inherent cellular plasticity within early cell types of the olfactory lineage, as well as potentially add to our knowledge of cellular reprogramming.

## Introduction

During early embryogenesis, pluripotent embryonic stem cells (ESCs) differentiate into the various specialized cell types that comprise the adult organism. This pluripotency is maintained by the expression of master regulatory transcription factors (TFs), including Sox2, Nanog, Oct4, Klf4, and Myc, which sustain gene networks associated with mitotic activity and poised chromatin states while repressing TFs that drive differentiation (Smith et al., 2016; Wang et al., 2021). The transition from pluripotency to lineage commitment is orchestrated by genetic and epigenetic mechanisms that establish and reinforce cell type-specific regulatory networks (Wutz, 2013). Epigenetics refers to heritable changes in gene expression that occur without alterations in the underlying DNA sequence, often mediated by modifications to chromatin structure and DNA methylation. A central concept of cellular differentiation is that epigenetic landscapes contribute to the stabilization of cell states.

Among the chromatin regulators implicated in cell fate determination, the Euchromatic histone-lysine *N*-methyltransferase 2 (*EHMT2*, also known as G9a) is an important chromatin factor that catalyzes the addition of local histone-3-lysine-9-methylation (H3K9me) marks that lead to locally silenced chromatin (Shankar et al., 2013). *EHMT2* plays a critical role in cell differentiation by silencing pluripotent factors (Eisenberg and Eisenberg, 2019), and disruption of *EHMT2* has been shown to facilitate induced pluripotency (Rodriguez-Madoz et al., 2017).

Cellular differentiation is typically unidirectional, and exceptions are mostly confined to atypical contexts such as tumor progression (Lee et al., 2016) or adult organ/tissue regeneration (Shoshani and Zipori, 2011). One documented case of apparent dedifferentiation unfolds under a regenerative regime within the adult mammalian olfactory system. Given its vulnerability to environmental insult, the olfactory epithelium (OE) requires continuous cellular replenishment throughout adult life (Graziadei and Graziadei, 1979; Kondo et al., 2010; Mackay-Sim and Kittel, 1991), representing one of the few known instances of mammalian neural regeneration (Murray and Calof, 1999). During a regenerative regime, a population of mitotically active basal cells differentiates into both neuronal and/or non-neuronal cell types (Gadye et al., 2017), the former involving a progression to committed neural progenitors (dGBC cells), immature olfactory sensory neurons (iOSNs), and finally mature olfactory sensory neurons (mOSNs) (Graziadei and Graziadei, 1979). Strikingly, injury-induced dedifferentiation of committed neural progenitors into multipotent stem cells has been observed (Lin et al., 2017), underscoring an ability of OE cells to overcome epigenetic barriers in response to severe injury, presumably as a hedging strategy to reallocate cellular resources.

Our laboratory utilizes the immortalized OP6 cell line (Illing et al., 2002; Pathak et al., 2009), which was previously characterized as an intermediate stage of the olfactory sensory neuronal (OSN) lineage. In this study, we interfered with *EHMT2* function to investigate OP6 cell stability, inspired by previous studies that implicate EHMT2 in OSN differentiation (Fiszbein et al., 2016; Lyons et al., 2014; Roopra et al., 2004). We found that *EHMT2* depletion caused transformation of OP6 cells to adopt altered cell morphology and growth characteristics, as well as upregulation of genes associated with the acquisition of pluripotency. These findings raise the possibility that differentiated cells along the OSN lineage might retain atypical plasticity in support of regenerative processes.

## Methods

### Cell culturing

The OP cell lines used in this study were previously described (Illing et al., 2002). For *EHMT2* knockdown experiments, we used CRISPR-Cas9 according to manufacturer protocols (Addgene). We designed sgRNAs targeting Exon 8 of the *EHMT2* gene, as well as a scrambled control sequence that should not target endogenous mouse loci. CRISPR/Cas9 lentiviral constructs were transiently transfected along with packaging plasmids pCMV-dR8.2dvpr and pCMV-VSV-G (Addgene) into HEK-293T cells (ATCC) using the standard Lipofectamine LTX transfection protocol (Invitrogen). OP6 cells were infected with harvested lentivirus and were analyzed and/or selected based on GFP expression from the lentiviral CRISPR-Cas9-eGFP delivery vector (Invitrogen). For some analyses, GFP+ cells were trypsinized and sorted using fluorescence-activated cell sorting. Colony frequencies were estimated by counting all GFP+ colonies per transfection against an estimate of total cell density in the dish. Colony growth rates were estimated using a standard curve that related maximum colony area to cell numbers derived from a confocal analysis of 16 sample colonies of various sizes. Immunocytochemistry was conducted on fixed cells and colonies using the following primary antibodies: rabbit anti-EHMT2 (C6H3) (Cell Signaling, 3306T, 1:200), anti-Oct4 (Abcam, ab184665, 1:200), anti-Klf4 (Abcam, ab106629, 1:200), anti-Nanog (Cell Signaling, 8822S, 1:200), anti-cMyc (Cell Signaling, 5605S, 1:200), anti-Sox2 (Abcam, ab92494, 1:200 dilution), and rabbit anti-Pax6 (Abcam, ab195045, 1:400). For Sox2 over-expression experiments, OP6 cells were transfected with the pCAG-HA-*Sox2*-IP plasmid (Addgene plasmid #13459) (Maruyama et al., 2005) using Lipofectamine LTX reagent and following standard protocols. Puromycin selection (4 µM) was employed to establish stable over-expression. Additional culturing, transfection, cell sorting, histochemistry, and colony sizing protocols are detailed within Supplementary Methods.

### Bulk RNA sequencing and single-nucleus RNA sequencing

For bulk sequencing, total RNA was isolated using Direct-zol RNA Microprep (Zymo Research) from various samples. For single-nucleus RNA sequencing, cell samples were frozen and sent to Novogene for library preparation, RNA sequencing, and bioinformatics using their standard protocols. The estimated number of nuclei per condition was 7588 for transformed colonies, 5040 for untransformed GFP+ cells with G9a perturbation (G9a_KO), and 12,314 for a mixture of wildtype OP6 cells and those treated with a scrambled guide RNA control (OP6_Scr). The total number of reads obtained per condition was 280,676,365 for transformed colonies, 250,702,146 for G9a_KO, and 250,185,958 for OP6_Scr. We conducted our own bioinformatics using 10X Genomic’s Cellranger pipeline (v 7.2.0), mapped with standard configuration onto a modified GRCm39 genomic annotation build. Mapped binary alignment map files were then count processed using velocyto v0.17.8 (La Manno et al., 2018) with rmasks enabled to output loom files. Scanpy v 1.9.4 (Wolf et al., 2018) was used for downstream processing. Data was first filtered on a minimum of 200 genes per cell and 2 cells per gene, then a second step removed low-quality cells if they expressed less than 600 genes. Predicted doublets were removed using Scrublet (Wolock et al., 2019). Integration of the three datasets was done using the bbknn algorithm built into Scanpy. RNA velocity was estimated using scvelo (La Manno et al., 2018) and the deterministic algorithm. Pseudobulk counts of each cluster were generated using the get_pseudobulk algorithm found in the decoupler package (Badia et al., 2022). Pseudobulking of single-nuclei RNA sequencing (snRNA-seq) data was performed to aggregate unique molecular identifier (UMI) counts within a specific cell cluster. This process involved summing UMI counts for each gene across individual cells within the cluster, followed by normalization of the total counts to 1 million.

## Results

We used the clonal OP6 cell line (Illing et al., 2002), which exhibits expression profiles consistent with a stage between a basal progenitor cell and mature olfactory sensory neuron (Illing et al., 2002; Pathak et al., 2009). OP6 cells can be induced with retinoic acid to differentiate into bipolar neurons that express more mature OSN (mOSN) marker proteins, as well as exhibit bipolar extensions characteristic of mOSNs *in vivo* (Pathak et al., 2009). Preliminary RNA-seq analyses on OP6 cells suggest that the founder cell for this line derived from an immature OSN (iOSN) of the olfactory placode, although expression of the immortalizing large T-antigen has caused expected drift in iOSN signature gene expression (Furuya et al., 2021). We have not previously observed any evidence of phenotypic variability in OP6 cultures.

We used a CRISPR-Cas9-eGFP cassette to interfere with a chromatin modifying factor, *EHMT2*, previously shown to be generally important for maintaining cellular states (Shinkai and Tachibana, 2011) (Fig. 1A). Bulk transcript analyses confirmed targeting of exon-8 of the *EHMT2* gene, where we identified a cluster of disruptive base deletions, with ∼half of EHMT2 transcripts exhibiting frame-shirt or deleterious mutations (Supplementary Fig. S1A). EHMT2 transcript and protein levels were reduced (Supplementary Fig. S1B and Fig. 1B, respectively), and H3K9me2 protein levels were decreased by 50% (Fig. 1C). Together, these results indicate that the CRISPR perturbation produced a knockdown phenotype.

**FIG. 1.**
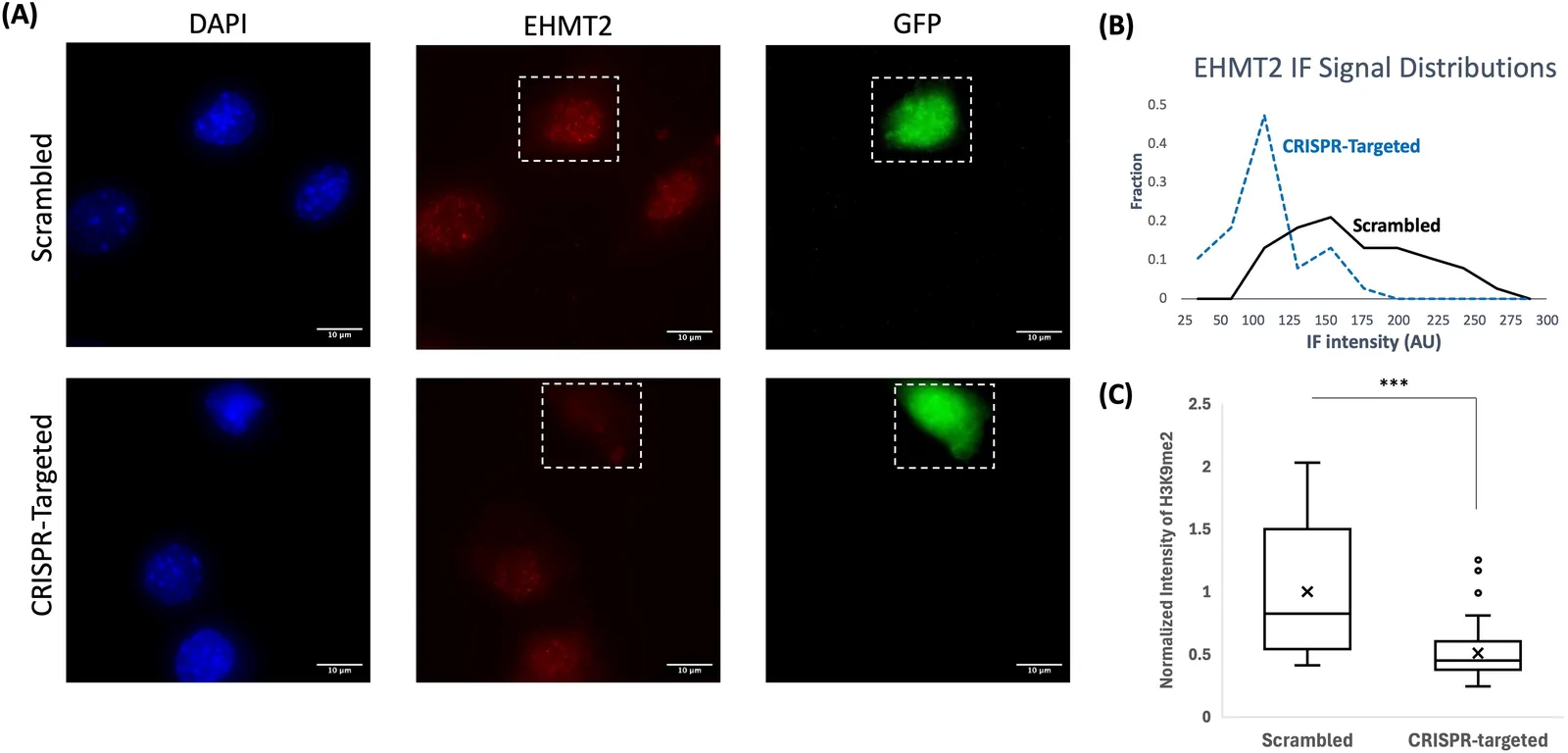
EHMT2 protein knockdown in CRISPR-treated cells. **(A)** Representative images showing EHMT2 expression (*red*) in scrambled (*top panel*) and CRISPR-targeted (*bottom panel*) samples. Hashed boxes highlight a green fluorescent protein (GFP)+ cell (*green*) in both the scrambled and targeted populations. Nuclei are counterstained with DAPI (*blue*). **(B)** Distribution of EHMT2 protein expression levels as measured by background-subtracted IF intensity within samples of scrambled control populations (*black*), *n* = 38 vs. untransformed GFP+ (CRISPR-targeted) cells (*blue*), *n* = 38. All cells exhibited expression above background noise levels. Mean IF intensity in the EHMT2-targeted sample is significantly reduced relative to scrambled control cells (*t*-test *p*-value = 10^−11^). **(C)** Box plot representing the distribution of normalized H3K9me2 immunofluorescence intensity for scrambled, *n* = 52 and untransformed GFP+ (CRISPR-targeted) cells, *n* = 58. Asterisks indicate statistical significance, where ***p-value < 0.00001. EHMT2, Euchromatic histone-lysine *N*-methyltransferase 2.

Bulk RNA-sequencing analysis of knockdown versus control samples revealed a small, but reproducible effect on the global transcriptome (*p* = 0.036). A preliminary Gene Ontology (GO) analysis on the ∼4% subset of differentially expressed genes (Supplementary Fig. S2A and B) identified functional enrichments in knockdown cells that included categories relating to cellular movement and migration, vascular formation, angiogenesis, tissue remodeling, and organogenesis, as well as extracellular matrix. Additionally, receptor-related terms signified potential enhancement of signal transduction and cellular communication processes. Together, these GO terms associated with EHMT2 knockdown suggest a complex molecular milieu, with the upregulation of specific processes that might be anticipated with changes in developmental status.

### Emergence of rare three-dimensional colonies within G9a-perturbed OP cell populations

To investigate the potential effects of EHMT2 knockdown on cellular behavior, we examined the growth characteristics of OP cells following CRISPR-Cas9-mediated G9a perturbation. After several days of post-transfection growth in normal OP cell culture media, we observed rare formation of three-dimensional colonies within knockdown cultures (Fig. 2A). The observed incidence was merely ∼5–10 colonies per million cells, or approximately ∼1 colony per 10,000 GFP+ cells. Similar colony formation was also observed following CRISPR-Cas9-mediated G9a perturbation in a second immortalized OP cell line, OP27, which has been previously characterized as a slightly less mature iOSN cell type (Illing et al., 2002) (Fig. 2B and C). In contrast, we never observed colony formation when CRISPR treatment of the immortalized GD25 cell line, a β1 integrin-deficient mouse fibroblast line immortalized via the SV40 large T-antigen (Wennerberg et al., 1996) (Fig. 2C), suggesting that colony formation might have depended on factors present within the olfactory lineage.

**FIG. 2.**
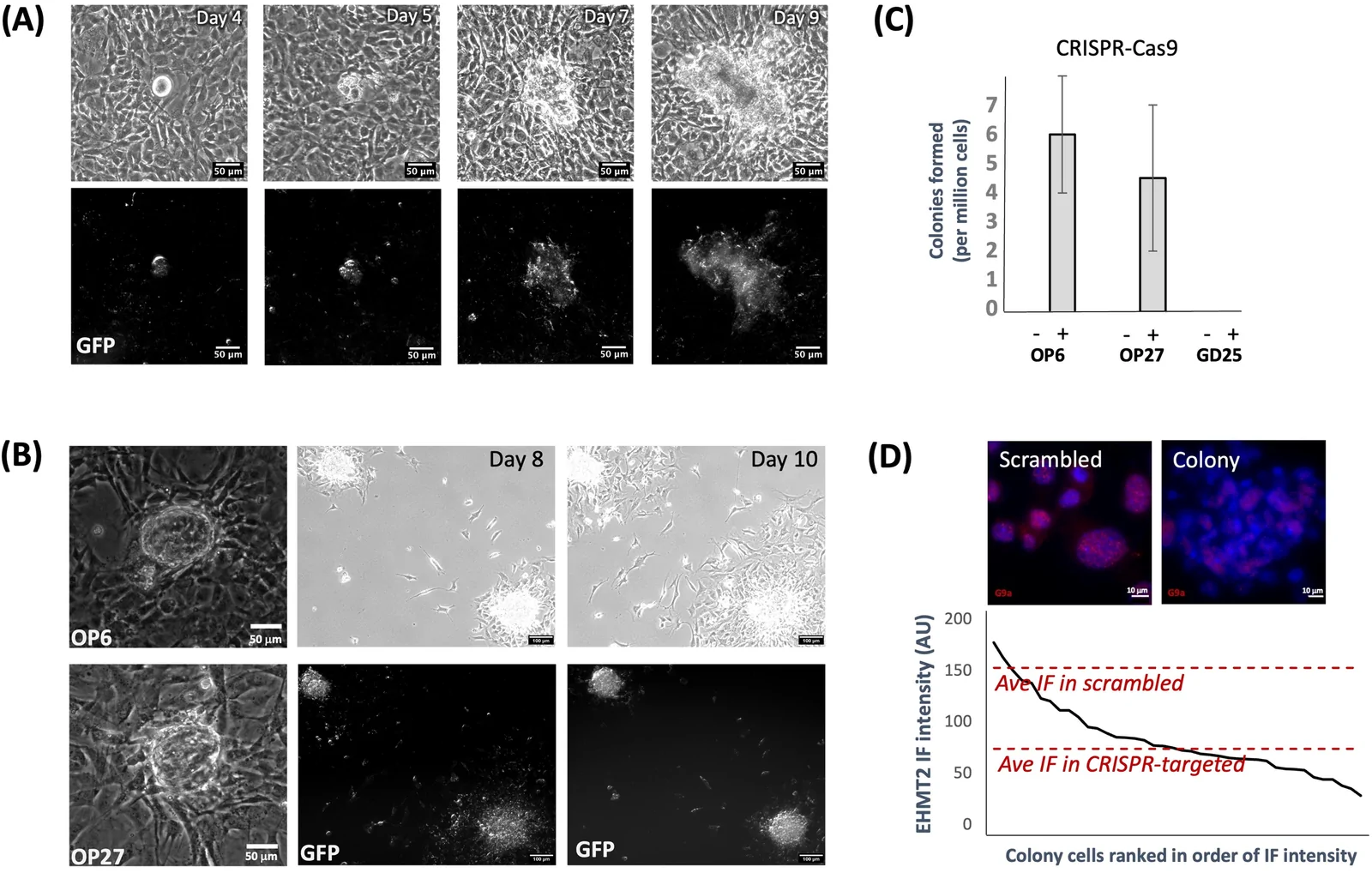
Rare colonies form in CRISPR-treated cultures. **(A)** Example of colony maturation from CRISPR-treated populations over a 9-day period. **(B)** *Left panels:* Example of a colony formed in treated OP6 and OP27 populations. *Right panels:* An example of a replated secondary colony after 9 days of growth (*top*) exhibiting maximum GFP fluorescence at the colony core (*bottom*). **(C)** Incidence of colony formation in three different immortalized cell lines (OP6, OP27, GD25) following CRISPR targeting of the EHMT2 gene (*n* = 5 biological replicates). **(D)** Immunofluorescence (IF) staining of EHMT2 (*red*) in control cells (*left*) vs. a representative colony (*right*). Nuclei are counterstained with DAPI (*blue*). The distribution of ranked EHMT2 IF intensities within colony cells (*n* = 40 random cells from seven representative colonies). Average IF intensity within scrambled cells (*upper line*) and sorted CRISPR-targeted cells (*lower line*) are indicated.

Primary OP colonies could be scraped and replated into secondary cultures, where they continued to grow (Fig. 2B). Notably, sorted single untransformed GFP+ cells (a homogeneous population of EHMT2-perturbed cells) failed to form colonies, even after extended culture. This observation hinted that colony formation might have required both wildtype and knockdown cells collaborating together. Perhaps consistent with this hypothesis, we noted that colonies consisted of both EHMT2-positive and negative cells (Fig. 2D).

### Colonies exhibit altered growth and cell morphological characteristics

We made several observations about colonies within EHMT2 knockdown cultures. First, we observed that colony growth was accelerated relative to the growth of wildtype OP6 cells (Fig. 3A, left panel). Second, colony growth rate decreased as cell density increased (Fig. 3A, middle and right panels). Density-dependent growth reduction could be due to competition for resources or contact inhibition (McClatchey and Yap, 2012). Third, colony formation and maintenance depended on ongoing expression of the large T-antigen (Fig. 3B). Fourth, we observed heterochromatin reorganization with the reduction of chromocenters (Fig. 3C); since chromocenter formation is dependent on H3K9me3 levels (Harničarová Horáková et al., 2010), this observation was consistent with reduced EMHT2 function. Fifth, we observed that many knockdown cells were more compacted and spherical in shape (Fig. 3D, left panels), as well as having a reduced average nucleus size (Fig. 3D, right panel). Each of the above phenotypic traits have been associated with more undifferentiated cellular states (Bongiorno et al., 2018; Butler et al., 2009; de Wit et al., 2013; Fussner et al., 2011; Koike et al., 2007; Meshorer et al., 2006; Wijchers et al., 2015; Ye et al., 2019), or transformation into a more tumor-like phenotype (Tellez et al., 2021; Thakur et al., 2022), although the latter might predict mitotic independence from the large T-antigen and/or loss of contact inhibition.

**FIG. 3.**
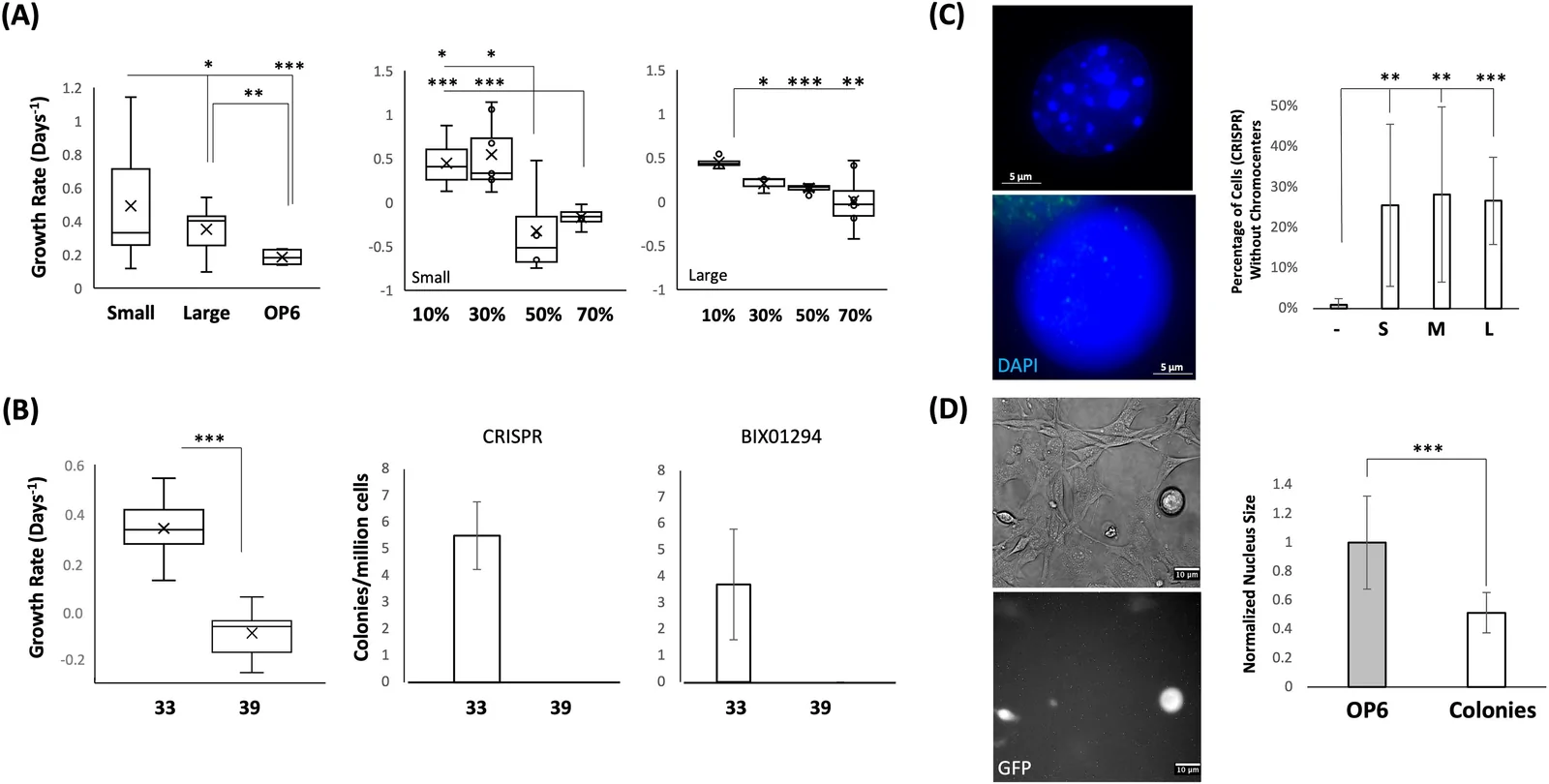
Colonies exhibit numerous altered cellular phenotypes. **(A)** *Left:* Growth rates are increased in small and large colonies relative to wildtype OP6 cells. *Middle and right:* Growth rates for small and large colonies at varying surrounding cell densities (*n* = 40 random cells from four representative colonies for each sample). **(B)** Colony growth rates (*left*) and colony formation (*middle* and *right*) cease when the large T-antigen is deactivated at the non-permissive temperature (39° C), (*n* = 4 biological replicates). **(C)** *Left:* The distribution of DAPI-dense staining becomes more uniform with apparent depletion of nuclear chromocenters. *Right:* Quantitation of the percentage of cells in wildtype (−), small (s), medium (m), and large (l) colonies that lack chromocenters (*n* = 30 random cells from four representative colonies for each sample). **(D)** *Left:* GFP+ cells commonly exhibit a more rounded cell shape relative to background non-transfected/GFP− cells. *Right:* CRISPR-targeted OP6 colonies exhibit reduced average nucleus size (*n* = 35 cells for each sample). Asterisks indicate statistical significance, where *p < 0.05, **p < 0.01, and ***p < 0.001.

### snRNA-seq reveals heterogeneous subpopulations within transformed OP6 colonies, including a stem cell-like cluster

We conducted snRNA-seq to investigate heterogeneity within transformed OP6 colonies. We learned during attempts to disperse colonies that they were resistant to single-cell isolations, presumably due to a stringent extracellular matrix. As a consequence, we were unable to use a typical cytoplasmic-based scRNA sequencing pipeline, and instead utilized a nucleus-based isolation strategy commonly employed for hard tissue samples (Guo et al., 2023). An integrated uniform manifold approximation and projection (UMAP) analysis identified two out of nine clusters, C3 and C8, that were predominantly composed of colony cells (Supplementary Fig. S3A and B), with C3 consisting exclusively of colony cells. Differential gene expression analysis revealed only six upregulated genes in C3 compared with all other clusters, including Cyclin Dependent Kinase 8 (CDK8), Calcium/Calmodulin Dependent Protein Kinase ID (Camk1d), and translation-associated genes Cmss1 and Lars2, as well as the Metallothionein 1 (Mt1) gene and the GM42418 cell differentiation lincRNA. Notably, four of these genes (CDK8, Camk1d, Cmss1, and Lars2) have been identified as putative regulators of pluripotent ESCs (Adler et al., 2012; Bergamaschi et al., 2008; Feng et al., 2022; Wang and Amoyel, 2022) (Supplementary Fig. S3C). This raises the possibility that the transformed colonies may have undergone a reversion toward a stem cell-like state.

We conducted a non-integrated UMAP analysis on three distinct cell populations: control (scrambled and WT OP6), sorted untransformed GFP+ cells, and isolated colonies (Fig. 4A and B). We found that colonies consisted of three subpopulations (termed C3, C4, and C5). The 104 genes that were significantly upregulated in the bulk colony sequencing dataset, as well as the set of 229 genes identified in bulk colony GO analyses, were likewise upregulated within pseudobulked C3, C4, and C5 subpopulations (Supplementary Fig. S4A and B), confirming consistency between the bulk and snRNA datasets.

**FIG. 4.**
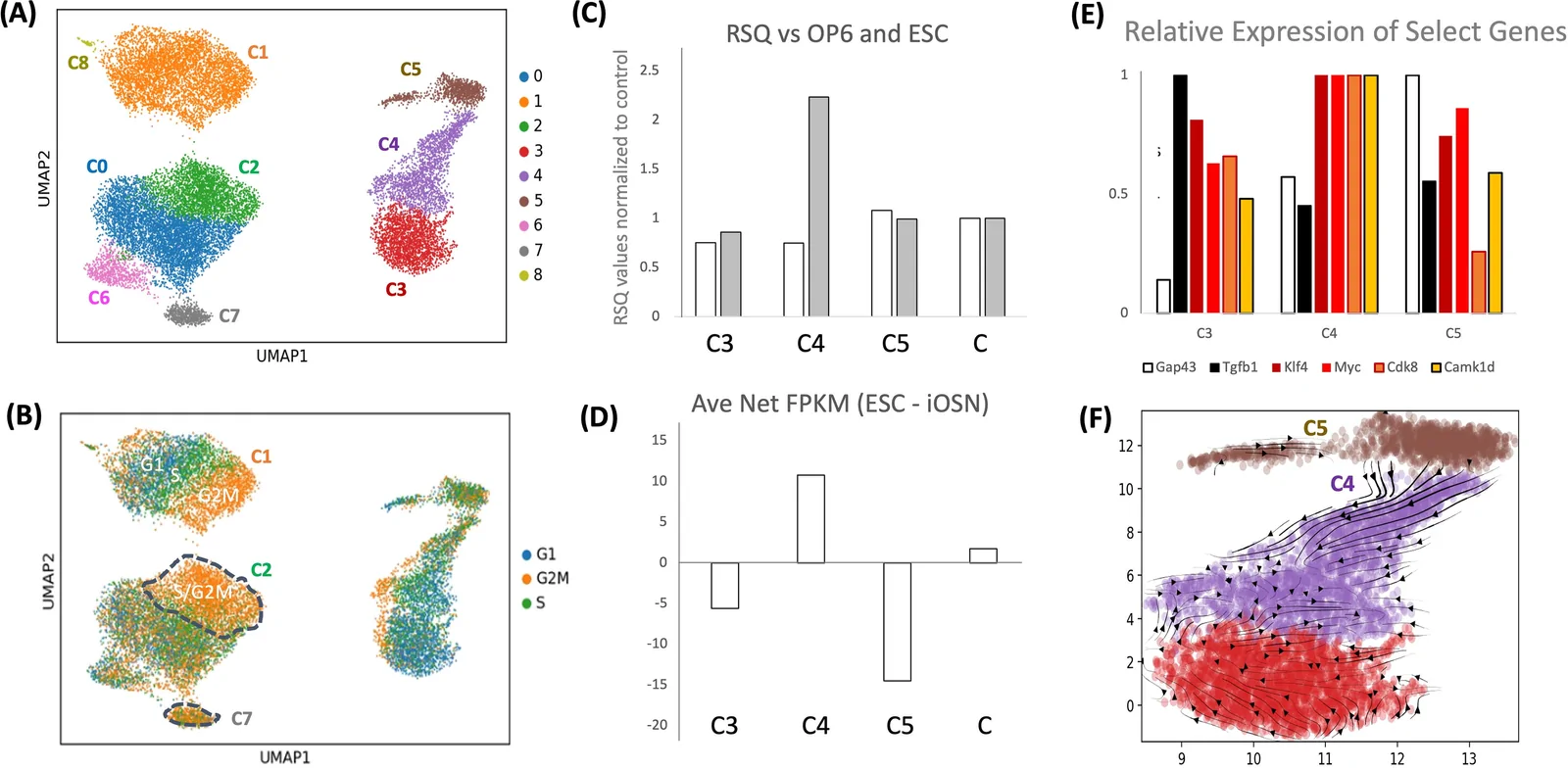
Single-cell analysis defines three colony subpopulations. **(A)** Non-integrated UMAP plots showing control subpopulations (C0, C2, C6, C7), sorted untransformed GFP+ subpopulations (C1, C8), and colony cell subpopulations (C3, C4, C5). The control sample consisted of a 50–50 mixture of scrambled CRISPR-treated OP6 cells and wildtype OP6 cells. The GFP+ cells indicated CRISPR-Cas9 expression of the EMHT2-targeting gRNA. **(B)** Population stratifications were partly accounted for by variable cell-cycle stages. **(C)** Transcriptome R-squared values (RSQ) between pseudobulked colony clusters (C3, C4, C5), as well as the average of the four control clusters (C = C0, C2, C6, C7) as compared with wildtype OP6 (*white bars*) and embryonic stem cell (ESC, *gray bars*) populations. **(D)** The average fragments per kilobase of transcript per million fragments mapped (FPKM) expression level in pseudobulked colony clusters (C3, C4, C5), as well as the average of the four control clusters (C = C0, C2, C6, C7), was calculated for a list of ESC-associated genes (EAGs) exhibiting >10-fold higher expression in ESCs vs. iOSNs with minimum FPKM = 100 in ESCs, and a list of iOSN-associated genes (OAGs) exhibiting >10-fold higher expression in iOSNs vs. ESCs with minimum FPKM = 100 in iOSNs (Kinoshita et al., 2021; Zunitch et al., 2023). The average net FPKM for each sample was then calculated as the difference between average EAG and OAG. A positive value for C4 indicates higher average expression levels for EAGs > OAGs; negative values for C3 and C5 indicate higher average expression levels for OAGs > EAGs. **(E)** Relative expression (normalized to maximum value for each gene = 1) for select genes in colony clusters (C3, C4, C5): the iOSN marker GAP43 (*open bar*), the signaling factor TGFB1 (*black*), two stem cell marker genes Klf4 (dark red) and Myc (*light red*), and two stem-associated factors, Cdk8 (*dark orange*) and Camk1d (*light orange*). **(F)** An RNA velocity analysis suggests transitions between colony C5 and colony C4 cells. List of genes can be found in Supplementary Data.

We tested whether any of these colony cell subpopulations were more OP6-like versus more ESC like (Kinoshita et al., 2021; Zunitch et al., 2023). We found that the pseudobulked C4 cell cluster was most strongly correlated with mouse ESC cells, whereas the C5 cell cluster was most strongly correlated with wildtype OP6 cells, both in terms of *r*^2^ values and signature gene expression (Fig. 4C and D), list of genes used for comparison can be found in Supplementary Data. We note that the C4 cluster exhibited upregulated RNA expression of two canonical ESC TFs, Klf4 and c-Myc, as well as expressing the highest levels of CDK8 and CAMK1D RNA (Fig. 4E), the former being involved with stem cell maintenance (Shinkai and Tachibana, 2011), and the latter involved in cell proliferation and epithelial-mesenchymal transitions (Bergamaschi et al., 2008). Perhaps interestingly, a key signaling molecule used within stem cell niches, TGFB1 (Xu et al., 2018) was upregulated in colonies and was maximally expressed in colony C3 cells, which, like C4 cells, were significantly diverged from wildtype OP6. Finally, RNA velocity analysis suggests transitions between colony C5 and C4 states (Fig. 4F). We consider each of these observations in a colony-forming model presented in the section “Discussion.”

### Colonies were upregulated in stem cell markers

Further to the hypothesis that transformed OP6 colonies might have adopted stem cell characteristics, we observed that transformed colonies showed elevated staining of all tested canonical marker proteins used to define pluripotent stem cell states (Fig. 5A and B). Moreover, these colonies stained for alkaline phosphatase (Fig. 5C), a marker for mouse ESCs (Andrews et al., 1984; Thomson et al., 1998). We also note that more mature (>200 cells) colonies exhibited heterogeneity in cell morphology, with more compacted, rounder cells evident at the core of the 3D mass, and a halo of more flattened cell monolayers at the periphery (Fig. 2B). This topology is reminiscent of embryoid bodies where differentiation occurs at the peripheral edges of stem cell derived colonies (Rosowski et al., 2015). Finally, we observed the down-regulation of the Pax6 neural lineage marker gene within colonies (Fig. 5A and B), which could signal loss of pre-existing neuronal states.

**FIG. 5.**
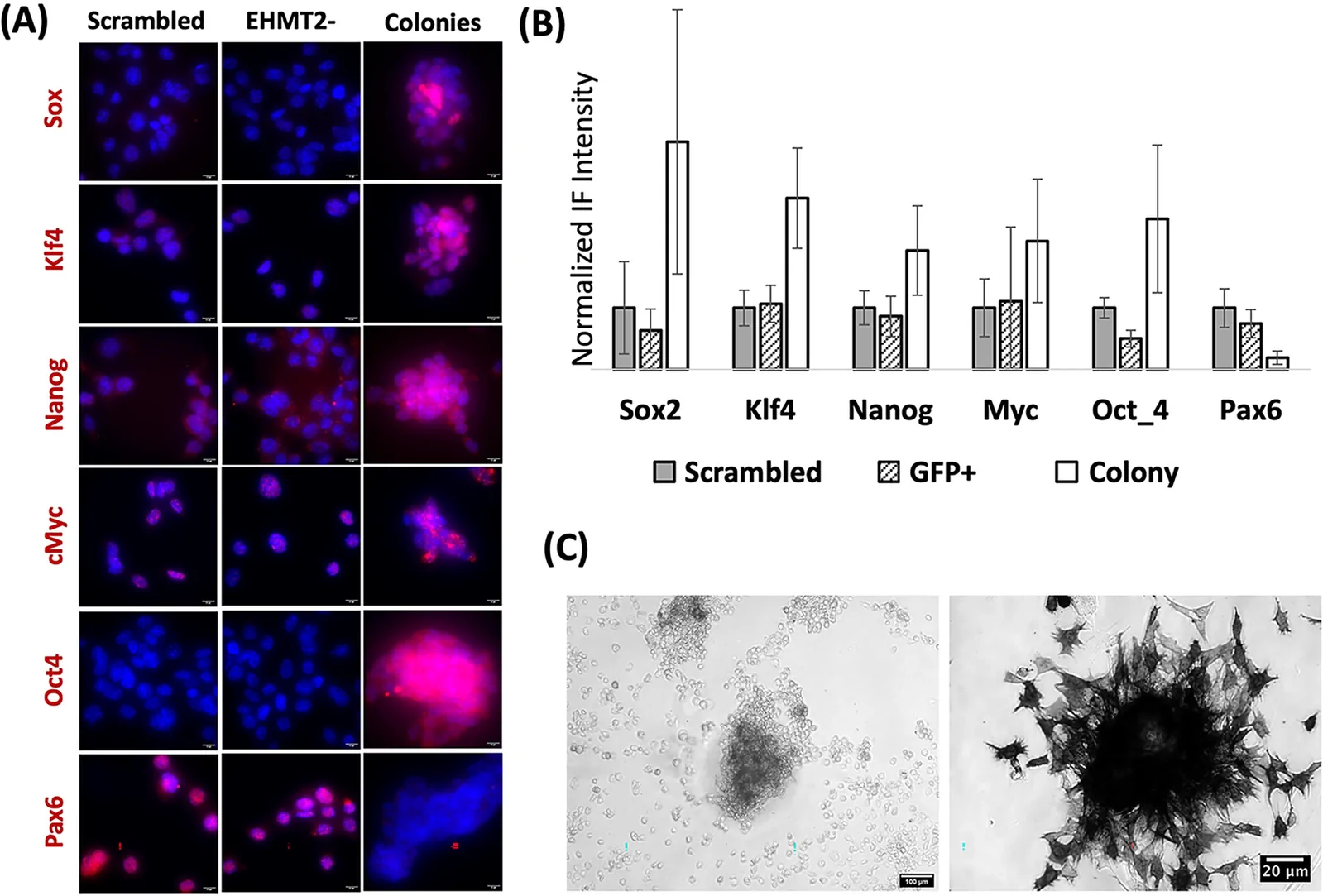
Immunofluorescence levels of canonical stem cell markers in G9a-perturbed OP6 colonies. **(A)** Upregulation of stem cell markers Sox2, Klf4, Nanog, cMyc, and Oct4 in CRISPR-derived OP6 colonies compared with scrambled control and untransformed GFP+ cells. Cell nuclei are visualized with DAPI staining (*blue*). Scale bars = 10 µm. **(B)** Bar graph summarizing the normalized immunofluorescence (IF) intensities of stem cell markers from the analysis of several CRISPR-derived OP6 colonies (+) compared with scrambled control and untransformed GFP+ cells. Intensities have been normalized to the average intensity in scrambled control. (*n* = 45 random cells from each sample) **(C)** Colonies stain with alkaline phosphatase, a marker of mouse ESCs.

### Sox2 over-expression synergistically contributes to OP6 colony formation

The rarity of colony formation suggested a stochastic attribute enabling transformation. One plausible stochastic attribute already hypothesized is the presumed rare occurrence of two nearby partner cells (*e.g.*, EHMT2+ and EHMT2−) that might be required for colony formation. Another possible stochastic variable is the presumed variable penetrance of CRISPR-mediated mutations, or other intrinsic factors known to be influential in cell reprogramming efficacy, such as cell-cycle stage or duration (Smith et al., 2010). We identified another potential source of stochastic variability that might have contributed to a low incidence of colony formation. As seen in Figure 6A, most wildtype OP6 cells, as well as knockdown cells, expressed very low levels of the Sox2 protein, a critical factor for establishing and maintaining pluripotency (Schaefer and Lengerke, 2020). However, we observed rare wildtype and knockdown OP6 cells with elevated Sox2 expression that approximated levels evident within colonies (Fig. 6A and B). We wondered whether rare Sox2 outlier cells might enable colony formation. We induced Sox2 over-expression in OP6 cells and observed colony formation with this treatment alone (Fig. 6C). When combining Sox2 over-expression with CRISPR-mediated EHMT2 perturbation, colony formation was ∼3.5 times enhanced relative to either treatment alone (Fig. 6C). These data, along with data already discussed, suggest that OP6 colony formation was enabled by three conditions: mitotic activity (*i.e.*, T-antigen expression, Fig. 3B), elevated expression of the Sox2 transcription factor, and reduction of chromatin barriers through EHMT2 perturbation.

**FIG. 6.**
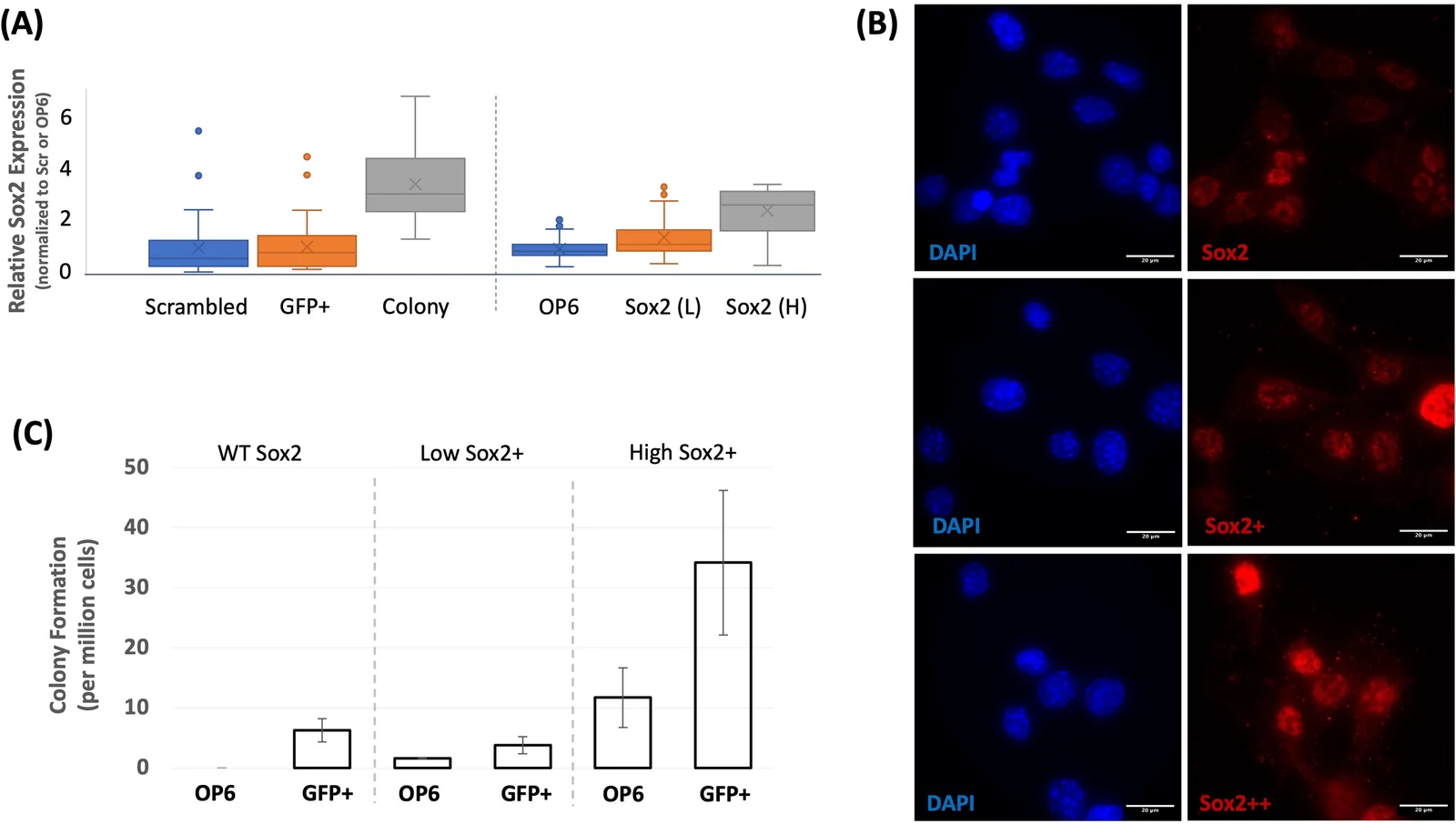
Sox2 expression enables colony formation in OP6 populations. **(A)** *Left:* Distribution of relative Sox2 antibody intensities in scrambled control cells (*blue*), untransformed GFP+ cells from primary transfected OP6 populations (*orange*), and within transformed colonies (*gray*). *Right*: Distribution of relative Sox2 expression in OP6 cells and two Sox2 transfections, one with lower overall Sox2 expression (l) and one with higher overall Sox2 expression (h), (*n* = 55 random cells from each sample). **(B)** Representative images showing Sox2 expression in wildtype OP6 cells (*top*), the low Sox2 over-expression experiment (*middle*), and the high Sox2 over-expression experiment (*bottom*). **(C)** Incidence of colony formation in wildtype OP6 cells versus EHMT2 perturbed GFP+ populations (*left panel*), in OP6 cells with Sox2 over-expression at lower levels, both with and without EHMT2 perturbation (*middle panel*), and in OP6 cells with Sox2 over-expression at higher levels, both with and without EMHT2 perturbation (*right panel*), *n* = 3 biological replicates.

## Discussion

We utilized CRISPR to knock down EHMT2 protein in olfactory-derived OP cells, and observed cell transformation based on several phenotypic markers, including cell morphology, growth properties, and gene expression status. One important question is whether this transformation was attributed specifically to EHMT2 perturbation. We reported targeted disruptive mutations in the EHMT2 gene, and that EHMT2 protein expression correspondingly decreased, and therefore, it seems unlikely that the observed phenotypes were due to a CRISPR off-target effect. To further validate, we independently targeted EHMT2 using the BIX01294 (Sigma) small molecule inhibitor, which occupies the EHMT2 histone binding site (Kubicek et al., 2007). We observed similar levels of OP colony formation within these BIX-drugged cultures (supplementary Fig. S5A); as expected, we also observed a reduction of H3K9me2/3 levels in these cells (Supplementary Fig. S5B). In addition, BIX-treated colonies upregulated the canonical stem marker proteins (Supplementary Fig. S5C). Therefore, two independent EHMT2 perturbations produced similar phenotypes. Finally, we note that immortalization via large T-antigen expression was necessary but not sufficient to cause transformation—colony formation does not occur without its expression, yet neither does it occur in wildtype OP cell lines (nor in the GD25 cell line) that are likewise immortalized (Fig. 3B). We propose that mitotic activity permitted EMHT2 perturbation to be effective —that is, progression through the cell cycle would be necessary to lose H3K9 methylation (Ma et al., 2015) and cell-cycle progression is generally a necessity for cell state change (Soufi and Dalton, 2016).

A second question concerns the rarity of colony formation. We note that under optimized protocols, ESC colony formation (iPSC) is also quite inefficient (Rao and Malik, 2012). We suggested three possible reasons for the low incidence of colony formation here involving stochastic attributes. First, we suggest that colony formation may require a rare partnership between a wildtype and knockdown cell, as suggested by the fact that colonies only form in mixed primary transfected cultures, but not within homogeneous EHMT2 knockdown cultures. Second, sox2 over-expression experiments suggest that colony formation depends on, or is enhanced by, elevated Sox2 levels, which are rare in wildtype OP cells. *In vivo*, stochastic Sox2 expression within the OSN lineage might constitute a bet-hedging strategy for reallocation of cellular resources within a regenerative regime. Dedifferentiation within the OE has previously been observed, and was enhanced by chromatin perturbation and compromised in a Sox2-depleted background (Lin et al., 2017). Third, transcriptional adaptation (TA) may limit transformation frequency following EHMT2 knockdown. TA involves the degradation of mutant or CRISPR-targeted mRNA and compensatory upregulation of homologous or functionally related transcripts (Sztal and Stainier, 2020). In this context, TA may partially restore G9a-dependent chromatin regulation through upregulation of paralogs such as G9a-like protein (Collins and Cheng, 2010). This buffering likely preserves epigenetic stability in most cells, restricting colony formation to rare instances where compensation fails.

### A model for colony formation

A third question we raise concerns the apparent cellular heterogeneity observed within colonies. As noted above, some of this heterogeneity might be attributed to a colony-forming partnership. We observed colony subpopulations with different cell morphologies and divergent gene expression profiles, including EHMT2 expression, that would be consistent with this idea. We speculate that EHMT2 knockdown in one cell may enable reactivation of ESC genes that might signal a partner wildtype cell to silence neural genes (requiring EHMT2 function). For example, the C3 colony subpopulation might have derived from a knockdown cell that subsequently upregulated TGFB1 to signal a nearby wildtype C5-like cell to dedifferentiate and upregulate stem markers, resulting in a more stem-like C4 cell. Perhaps consistent with this model, we note that an RNA-seq trajectory analysis suggested transitions from C5 (most OP6-like) to C4 (most stem-like).

The most important question of our study pertains to whether some of the cells within colonies had indeed reprogrammed toward a pluripotent stem cell. We have described several provocative indicators, including canonical stem cell marker upregulation. We are unable to confidently map snRNA-seq profiles onto previously characterized iOSN and ESC datasets to more rigorously address this question because expression of the large T-antigen (Deng et al., 2020) and nuclear mRNA sourcing (Kim et al., 2023) both broadly impact transcript representation. Nevertheless, we are motivated in future studies to utilize strategies commonly employed for validating iPSCs both *in vitro* and *in vivo* (Cerneckis et al., 2024). Should transformed OP6 cells meet established criteria in the field for iPSC status, our work would provide additional momentum for exploring the regenerative environment within the olfactory system as a possible new source for stem cells with neuronal origins.
