# Supplementary material for: Transformation of an Olfactory Placode-Derived Cell into One with Stem Cell Characteristics by Disrupting Epigenetic Barriers
Source: Cell Reprogram. 2025 Aug 12;27(4):164–73. doi: 10.1177/21524971251359000 (PMC13622967; doi:10.1177/21524971251359000)
Supplement: Supplementary file 1 [file 21524971251359000_SupplementaryDataS2.docx]

**Supplementary Methods**

**Cell culturing.** OP6 cell lines were grown at the permissive temperature for the large T-antigen (33^0^C) in DMEM (Gibco) supplemented with 10% FBS (Gibco). Details for buffers, growth conditions, and passaging protocols are available in Noble et al. (2018). The GD25 cell line (Wennerberg et al., 1996), 293T cell line (DuBridge et al., 1987), and the mouse embryonic stem cell line (Robertson et al., 1986) was cultured according to published protocols. In some experiments, we grew cells in media supplemented with 1 mM or 2 mM of the drug BIX01294 EHMT2 inhibitor (Sigma). Cell densities within experimental cultures were determined by counting cells within a 1180 μm² area in brightfield images, with cell density calculated as the average of 10 randomly selected fields per dish. The total cell count was then extrapolated based on the calculated cell density across the entire dish.

**CRISPR-Cas9 gene targeting.** We utilized CRISPR-Cas9 according to manufacturer protocols (Addgene). To target Exon 8 of the EHMT2 gene, we designed and annealed complementary oligonucleotides 25 bp in length, and cloned the guide sequence into the lentiviral CRISPR-Cas9-eGFP delivery vector (Invitrogen). Following sequencing verification, CRISPR-Cas9 lentiviral contstructs were transiently transfected along with packaging plasmids pCMV-dR8.2dvpr and pCMV-VSV-G (Addgene) into HEK-293T cells (ATCC) using standard Lipfectamine LTX transfections (Invitrogen). Approximately 10^5^ host OP6 cells were subjected to infection at RT in the presence of 8 ug/ml polybrene as described elsewhere (Stewart et al., 2003).

**Colony growth rate estimates.** For some experiments, colony growth rates in living colonies were estimated using a standard curve that related maximum cross-sectional colony area to cell numbers, which were derived from a confocal analysis of 16 sample colonies of various sizes (ranging from 12 to 105 cells). The standard curve derived from this analysis is shown below.

Standard Curve

Area (um2)

| Cell Number | Area |
| --- | --- |
| 92 | 9130.9 |
| 62 | 5491.17 |
| 65 | 8375.53 |
| 32 | 3673.1 |
| 15 | 2662.6 |
| 17 | 2462.97 |
| 84 | 9734.47 |
| 27 | 4336.62 |
| 43 | 5722.14 |
| 44 | 4489.74 |
| 82 | 6925.09 |
| 12 | 1728.28 |
| 105 | 11045.9 |
| 34 | 5269.72 |
| 33 | 5639.72 |
| 54 | 7985.04 |

Growth rates (gr) of colonies under various experimental conditions were calculated using:

*gr* =

ln (N_t_ / N_0_)

*t*

where N_t_ is the number of cells at time *t*, N_0_ is the initial number of cells, and *t* is the elapsed time (Singer, 1986).

**Fluorescence-activated cell sorting (FACs) of GFP+ cells.** Four days post-transfection, OP6 cells transfected with CRISPR-Cas9-eGFP targeting G9a or a scrambled control guide RNA were harvested via trypsin dissociation, washed twice with PBS, and filtered through a 100 µm mesh. Cells were resuspended in harvesting medium (PBS with 1 mM EDTA, 25 mM HEPES pH 7.0, and 2% heat-inactivated FBS) at a concentration of 10 million cells/mL. FACS was performed on a BD FACSymphony S6 SE, sorting 100,000 GFP+ cells in bulk and conducting single-cell sorting into collection buffer (DMEM with 30% heat-inactivated FBS, 1X antibiotic-antimycotic, and 25 mM HEPES pH 7.0).

**Immunocytochemistry and alkaline phosphatase staining.** Cells and colonies were fixed in 3% paraformaldehyde for 10 minutes and 8% paraformaldehyde for 30 minutes, in PBS buffer containing 0.5% Triton-X (Sigma). Fixed cells were blocked with 1% BSA and 0.1% Tween-20. The following primary antibodies were used in this study: rabbit anti-EHMT2 (C6H3) (Cell Signaling, 3306T, 1:200), anti-Oct4 (Abcam, ab184665, 1:200), anti-Klf4 (Abcam, ab106629, 1:200), anti-Nanog (Cell Signaling, 8822S, 1:200), anti-cMyc (Cell Signaling, 5605S, 1:200), anti-Sox2 (Abcam, ab92494, 1:200 dilution) rabbit anti-Pax6 (Abcam, ab195045, 1:400). The following secondary antibodies were used: donkey anti-mouse-Cy3 (Jackson Immunoresearch, 715-165-150, 1:100), goat anti-mouse Alexa 488 (Abcam, ab150117, 1:200), donkey anti-rabbit Alexa 488 (Jackson Immunoresearch, 711-545-152, 1:100), and goat anti-rabbit-Cy3 (Millipore, AP132C, 1:800). Immunofluorescence images were acquired using a Deltavision RT imaging system (Applied Precision) adapted to an Olympus (IX71) microscope equipped with XYZ motorized stage. Images were processed using Softworx (Applied Precision) or FIJI (ImageJ). Individual nuclei were optically sectioned at 0.5 mm intervals to resolve spatial signal patterns, and in some cases, these confocal images were projected onto a single plane for visualization. Immunofluorescence intensities were quantified in absolute units using FIJI (ImageJ) after background subtraction. Alkaline phosphatase staining was conducted on primary OP6 cell cultures using NBT/BCIP stock solution (Roche/Sigma 11681451001) or BCIP/NBT-Blue Liquid Substrate Systems for Membranes (Sigma B3804) in accordance with manufacturer’s protocols.
