# Supplementary material for: Transformation of an Olfactory Placode-Derived Cell into One with Stem Cell Characteristics by Disrupting Epigenetic Barriers
Source: Cell Reprogram. 2025 Aug 12;27(4):164–73. doi: 10.1177/21524971251359000 (PMC13622967; doi:10.1177/21524971251359000)
Supplement: Supplementary file 2 [file 21524971251359000_SupplementaryFigureS1.docx]

**Supplementary Figure 1. EHMT2 gene targeting and protein knockdown. (A)** Bar graph illustrating the percentage of bulk RNA-seq reads for different types of mutations detected within the CRISPR-targeted EHMT2 nucleotide sequence in the vicinity of the guide RNA location. Portions of bars colored blue depict deletions, green depict insertions, and other colors depict mutated bases. The corresponding region within the EHMT2 gene in the scrambled CRISPR control did not exhibit mutational clustering and suggested a background mutational frequency of ~100-fold lower than observed in the EMHT2 targeted sample. **(B)** EHMT2 transcript levels in CRISPR-targeted versus control samples at single-cell resolution. Violin plots illustrating distributions of EHMT2 transcript levels from pseudobulked targeted and control samples (right). The control sample used for snRNA-seq was a ~50-50 mixture of cells treated with a scrambled guide RNA cassette and wildtype OP6 cells.
