# Supplementary material for: Transformation of an Olfactory Placode-Derived Cell into One with Stem Cell Characteristics by Disrupting Epigenetic Barriers
Source: Cell Reprogram. 2025 Aug 12;27(4):164–73. doi: 10.1177/21524971251359000 (PMC13622967; doi:10.1177/21524971251359000)
Supplement: Supplementary file 3 [file 21524971251359000_SupplementaryFigureS2.docx]

**Supplementary Figure 2. Transcriptomic Alterations Following EHMT2 CRISPR-Cas9 Targeting. (A)** Volcano plot combining two independent CRISPR-Cas9 experiments targeting the EHMT2 gene versus scrambled control samples depicting up-regulated (red) and down-regulated (green) genes exhibiting >2-fold change in experimental versus control samples. Minus log10 p-values are plotted on the vertical axis. **(B)** Gene Ontology (GO) analysis of differentially expressed genes in the bulk CRISPR-targeted sample relative to scrambled control.
