# Supplementary material for: Transformation of an Olfactory Placode-Derived Cell into One with Stem Cell Characteristics by Disrupting Epigenetic Barriers
Source: Cell Reprogram. 2025 Aug 12;27(4):164–73. doi: 10.1177/21524971251359000 (PMC13622967; doi:10.1177/21524971251359000)
Supplement: Supplementary file 4 [file 21524971251359000_SupplementaryFigureS3.docx]

**Supplementary Figure 3. Bioinformatic analysis of snRNA-seq data conducted by Novogene**. **(A)** UMAP plot integrating snRNA-seq data from three sample groups: transformed colonies, untransformed OP6 cells with G9a perturbation (G9a_KO), and a mixture of wildtype OP6 cells and those treated with a scrambled guide RNA control (OP6_Scr). Clustering analysis identified nine distinct cell populations, with Cluster 3 consisting exclusively of colony-associated cells. Inset: UMAP plot color-coded by cluster composition, highlighting the distribution of colony cells (red) across multiple clusters. **(B)** Quantification of cluster composition across samples, showing the proportional representation of each cluster within each sample. Notably, Cluster 3 is composed entirely of colony cells, while Cluster 8 is predominantly colony-associated. **(C)** Differential gene expression analysis identified six genes significantly upregulated in Cluster 3 compared to all other clusters, with the same genes also exhibiting upregulation in Cluster 8.
