# Supplementary material for: Transformation of an Olfactory Placode-Derived Cell into One with Stem Cell Characteristics by Disrupting Epigenetic Barriers
Source: Cell Reprogram. 2025 Aug 12;27(4):164–73. doi: 10.1177/21524971251359000 (PMC13622967; doi:10.1177/21524971251359000)
Supplement: Supplementary file 5 [file 21524971251359000_SupplementaryFigureS4.docx]

**Supplementary Figure 4. Colony signature genes up-regulated in all three pseudubulked colony subpopulations. (A)** Average FPKM expression levels within pseudobulked clusters (C0-C8) from nucleus-derived RNA for a set of genes that are >4-fold up-regulated (n=101 genes) in bulk colony populations relative to untransformed GFP+ populations. **(B)** Average FPKM expression levels within pseudobulked clusters (C0-C8) from nucleus-derived RNA for sets of genes identified as significantly enriched GO terms in bulk colony populations: upper green = endothelial (n=17 genes), lower green = epithelial (n=25), upper blue=angiogenesis (n=29), lower blue=cartilage (n=12), upper orange=cell adhesion (n=23), middle orange=cell migration (n=34), lower orange=ECM (n=30), upper black=skeletal (n=24), next black=muscle development (n=9), next black=mammary development (n=10), lower black=neuro development (n=16). The solid red line indicates the average for FPKM for all 229 of these genes. By both metrics, the C3 subpopulation best captures bulk gene up-regulation trends that define bulk colony transitions.
