# Supplementary material for: Transformation of an Olfactory Placode-Derived Cell into One with Stem Cell Characteristics by Disrupting Epigenetic Barriers
Source: Cell Reprogram. 2025 Aug 12;27(4):164–73. doi: 10.1177/21524971251359000 (PMC13622967; doi:10.1177/21524971251359000)
Supplement: Supplementary file 6 [file 21524971251359000_SupplementaryFigureS5.docx]

**Supplementary Figure 5. Colony transformation in BIX-treated OP6 and OP27 cells. (A)** Colony forming incidence with BIX treatment of the immortalized OP6, OP27, and GD25 cell lines (n=4 biological replicates). **(B)** Western blot of whole cell lysate using antibodies against actin, histone H3, dimethylated H3 (H3K9me2), and trimethylated H3 (H3K9me3) proteins in untreated OP6 cells (0 lanes), and with 2 um (2 lanes) and 4 um (4 lanes) BIX inhibitor drug to the EHMT2 protein. **(C)** Immunofluorescence images of mouse embryonic stem cells (mESC) untreated OP6 cells, and BIX-treated OP6 colonies showing the expression of Sox2, Klf4, Nanog, cMyc, and Oct4 stem cell markers. Cell nuclei are visualized with DAPI staining (blue). Scale bars = 10 μm. *Lower*: Bar graph depicting the fold-increases of Sox2, Klf4, Nanog, cMyc, and Oct4 within BIX-treated OP6 cells (+) relative to untreated controls (-) (n=35 cells).

For Western blots OP6 cells were at either 2 or 4 µM of BIX compound for 4 days. Following trypsinization and washing, cell pellets were resuspended in Radio-Immunoprecipitation Assay (RIPA) lysis buffer supplemented with 10x protease inhibitor cocktail per manufacturer’s protocols. Supernatants containing protein lysates were quantified, denatured, and transferred from SDS gel to PVDF membrane. Following washing with 0.1% Tween-20 in TBST, membranes were blocked with 5% BSA and incubated with 1:5000 rabbit anti-H3 (Abcam, catalog number ab1791), 1:2000 mouse anti-H3K9me2 (Abcam, catalog number ab1220), 1:2000 rabbit anti-H3K9me3 (Millipore, catalog number 07-442), or 1:5000 rabbit anti-Actin (Abcam, catalog number: Ab8227). Western blot bands were visualized using 1:10,000 donkey anti-rabbit alkaline phosphatase (Abcam, catalog number ab97061), 1:1000 mouse anti-rabbit alkaline phosphatase (Sigma, catalog number A2556) or 1:1000 goat anti-mouse alkaline phosphatase (Sigma, catalog number A3562).
